# Trends in health services utilization, medication use, and health conditions among older adults: a 2-year retrospective chart review in a primary care practice

**DOI:** 10.1186/1472-6963-9-217

**Published:** 2009-11-30

**Authors:** Ketan Vegda, Jason X Nie, Li Wang, C Shawn Tracy, Rahim Moineddin, Ross EG Upshur

**Affiliations:** 1 Primary Care Research Unit, Sunnybrook Health Sciences Centre, 2075 Bayview Ave, Room E3-49, Toronto, ON M4N 3M5 Canada; 2 University of Toronto Joint Centre for Bioethics, 88 College Street, Toronto, ON M5G 1L4 Canada; 3 Institute for Clinical Evaluative Sciences, 2075 Bayview Ave., Toronto, ON M4N 3M5 Canada; 4 Department of Family and Community Medicine, Faculty of Medicine * University of Toronto, 263 McCaul Street, 3rd Floor, Toronto, Ontario, M5T 1W7 Canada; 5 Department of Public Health Sciences, University of Toronto, Building, 155 College Street, Toronto, ON M5T 3M7, Canada

## Abstract

**Background:**

Population aging poses significant challenges to primary care providers and healthcare policy makers. Primary care reform can alleviate the pressures, but these initiatives require clinical benchmarks and evidence regarding utilization patterns. The objectives of this study is to measure older patients' use of health services, number of health conditions, and use of medications at the level of a primary care practice, and to investigate age- and gender-related utilization trends.

**Methods:**

A cross-sectional chart audit over a 2-year study period was conducted in the academic family practice clinic of Sunnybrook Health Sciences Centre in Toronto, Ontario, Canada. All patients 65 years and older (n = 2450) were included. Main outcome measures included the number of family physician visits, specialist visits, emergency room visits, surgical admissions, diagnostic test days, inpatient hospital admissions, health conditions, and medications.

**Results:**

Older patients (80-84 and 85+ age-group) had significantly more family physician visits (average of 4.4 visits per person per year), emergency room visits (average of 0.22 ER visits per year per patient), diagnostic days (average of 5.1 test days per person per year), health conditions (average of 7.7 per patient), and medications average of 8.2 medications per person). Gender differences were also observed: females had significantly more family physician visits and number of medications, while men had more specialist visits, emergency room visits, and surgical admissions. There were no gender differences for inpatient hospital admissions and number of health conditions. With the exception of the 85+ age group, we found greater intra-group variability with advancing age.

**Conclusion:**

The data present a map of greater interaction with and dependency on the health care system with advancing age. The magnitudes are substantial and indicate high demands on patients and families, on professional health care providers, and on the health care system itself. There is the need to create and evaluate innovative models of care of multiple chronic conditions in the late life course.

## Background

The aging of the baby-boom generation poses a serious challenge to healthcare providers and policy planners. It is projected that on a global level between 2000 and 2030, the ≥65 year old population will increase from 6.9% to 12.0% [1]. The development of disease-modifying therapies enables individuals to survive longer, but with higher prevalence of multiple chronic diseases and increased preventive medications [2]. The demographic reality raises some challenging questions about whether the infrastructure and human resources are in place to care for this coming cohort, and whether the present Canadian healthcare model is optimal for chronic disease management. At present in Canada, the publicly-funded healthcare system is best described as an interlocking set of ten provincial and three territorial health insurance plans, each of which provides access to universal, comprehensive coverage for medically necessary hospital and physician services.

In an era when patient-centered care and disease-specific guidelines vie for greater consideration, we need to understand the current situation of chronic disease management in the primary care context, as it is the most common setting for the provision of chronic disease care [3-5]. A number of previous studies have explored health services utilization and morbidity patterns by age and gender [6-13]. However, much of the available data classifies all those aged 65 and above as one homogeneous group, lack a primary care focus, have been predominantly disseminated to gerontology and geriatrics audiences, and examine only one particular health service or one specific disease condition. Although it is well know that utilization increases with advancing age, the current study aims to add precision to the existing knowledge by using a large patient sample with close measurements of five-year age groups of all older patients in a family practice unit. The objective of this study is to investigate patterns of use of health services among patients aged 65+ in a defined academic family practice setting.

## Methods

### Study Design

We conducted a retrospective chart audit for all patients 65 years old and over in the Department of Family and Community Medicine (DFCM) of Sunnybrook Health Sciences Centre (SHSC).

### Setting and Study Population

The Sunnybrook DFCM is an academic primary care clinic in Toronto, Ontario, Canada using a health services organization (HSO) practice model since 1983. Physicians are on salary, and not fee-for-service, and receive moneys per patient, per month based on age and sex, not on the number of times that a particular patient is seen. The clinic has 13 staff physicians, 24 residents, and 10 nurses. It provides care to approximately 8500 patients, with a focus on older patients with complex chronic diseases. All patients (N = 2731) who had at least their 65th birthdays during 2004 were included in the study. Health service utilizations in the two-year period of September 1, 2004 to August 31, 2006 were collected.

### Variables of Interest

Demographic information, such as gender, age, and marital status were collected from the Cumulative Patient Profile (CPP). Eight outcome variables were selected: family physician visits, specialists visits, emergency room visits, test days, surgical admissions, in-patient hospital admissions, medication use, and chronic health conditions from the patients' CPP. Family physician (FP) visits included all visits made to the DFCM, excluding missed appointments and phone calls. The number of specialist visits (SP) was determined by letters from specialists to the family physician and referral forms from family physicians to specialists. The number of emergency room (ER) visits was counted using emergency room reports appearing in the chart. Test days on which blood and diagnostic tests were performed were counted (multiple tests performed on a single day were counted as one test day). Endoscopies and colonoscopies were included as diagnostic tests. Surgical admissions included the number of times a patient was admitted to a hospital for surgery. In-patient hospital admissions were determined by the number of times a patient was admitted to the hospital for reasons other than surgery or visits to an emergency room as determined by discharge summaries in the chart. Data on medication use and chronic health conditions were collected from the patients' CPP. Any additional medications and chronic health conditions not listed in the CPP were identified from patient chart entries for the two-year study period.

### Statistical Analysis

Patients were sorted by gender and age into groups of 5-year intervals as determined by their age on January 1, 2005. Records were manually abstracted and entered by trained chart abstractors into a Microsoft Access database. Descriptive statistics were calculated for each variable by age and gender. Statistical significance between genders within an age group was assessed using one-way ANOVA at p = 0.05 level. To compare means across age groups within a gender, we used the Tamhane post-hoc method assuming unequal variances at the p = 0.05 and p = 0.001 levels. Data was analyzed using SPSS (v11.0.1, 2001).

The study was approved by the Sunnybrook Health Sciences Centre Research Ethics Board.

## Results

Following data cleaning (where errors in data entry were screened and corrected, and records with insufficient information and duplicate entries were removed), a total of 2,450 valid patient records remained (Table 1). While the chart auditors made repeat attempts to obtain patient charts that were missing at the time of the audit (e.g. in use by the clinic), there were still approximately 280 charts that were not audited as the charts were either not available or lost. It is difficult to determine the effect of these missing charts on our results, which may lead to an under- or over-estimation. There were more females in the family practice unit than males, and more who were married than not. Most patients fell in the 75-79 age-group.

**Table 1:** Demographics of the patient population 65 years and older in the Department of Family and Community Medicine of Sunnybrook Health Sciences Centre

		Age group (years)					
			
		65-69 n (%)	70-74 n (%)	75-79 n (%)	80-84 n (%)	85+ n (%)	Total n (%)
**Gender**							
	Men	204 (8)	223(9)	252 (10)	198 (8)	105 (4)	982 (40)
	Women	274 (11)	314 (13)	317 (13)	311 (13)	252 (10)	1468 (60)

	**Total**	478 (19)	537 (22)	569 (23)	509 (21)	357 (14)	2450 (100)

**Marital Status**							
	Married	332 (14)	365 (15)	336 (14)	250 (10)	116 (5)	1399 (57)
	Not Married	146 (6)	172 (7)	233 (10)	259 (11)	241 (10)	1051 (43)

	**Total**	478 (20)	537 (22)	569 (23)	509 (21)	357 (15)	2450 (100)

Table 2 summarizes the annual rates of health services use and number of chronic health conditions and medications, by gender and age group. Among the types of health services utilized, blood and diagnostic test days had the highest counts with an average of 5.1 test days per person per year. The oldest (85+) group had 34% more test days than the youngest (65-69) group. The variability (standard deviation) was also greater among the three older age groups. There was no overall difference between men and women.

**Table 2:** Annual rates of health services utilization, and number of chronic health conditions and medications listed in patient charts during 2-year period ending August 31, 2006.

Indicator	Statistic	Women						Men						Overall					
		
		65-69	70-74	75-79	80-84	85+	Overall	65-69	70-74	75-79	80-84	85+	Overall	65-69	70-74	75-79	80-84	85+	Overall
**FP Visits**	Mean	3.8*	4.1	4.8^‡^	4.9^‡^	5.5*^‡^	4.6*	3.2	3.8	4.3^‡^	5.1^‡^	4.5^‡^	4.2*	3.6	4	4.6	5	5.2	4.4
	Median	3.5	3.5	4	4.5	5	4	2.5	3.5	3.5	4.5	4	3.5	3	3.5	4	4.5	4.5	4
	Std	2.3	2.8	3.4	3.3	3.3	3.1	2.5	2.8	2.7	3.4	2.5	2.9	2.4	2.8	3.1	3.3	3.1	3

**SP Visits**	Mean	1.9*	2.1	2.4*	2.5^†^	1.9	2.2*	2.4*	2.4	3.1*	2.9	2.4	2.7*	2.1	2.2	2.7	2.7	2.1	2.4
	Median	1.5	1.5	1.5	2	1	1.5	1.5	1.5	2.5	2	2	2	1.5	1.5	2	2	1.5	1.5
	Std	2	2.3	2.5	2.5	2.4	2.4	2.8	2.3	2.9	2.9	2.2	2.7	2.4	2.3	2.7	2.6	2.3	2.5

**ER Visits**	Mean	0.12	0.13*	0.23^†^	0.28^‡^	0.31^‡^	0.21	0.16	0.21*	0.25	0.30^†^	0.35^†^	0.24	0.14	0.16	0.24	0.28	0.32	0.22
	Median	0	0	0	0	0	0	0	0	0	0	0	0	0	0	0	0	0	0
	Std	0.28	0.28	0.44	0.6	0.57	0.46	0.43	0.57	0.45	0.46	0.53	0.49	0.35	0.43	0.44	0.55	0.56	0.47

**Diagnostic Test Days**	Mean	4.4	4.5	5.5^†^	5.4*^†^	5.6^†^	5.1	3.9	4.5	5.5^†^	6.6*^‡^	5.8^†^	5.2	4.2	4.5	5.5	5.9	5.7	5.1
	Median	3.8	3.5	4	4	4.5	4	3	3.5	4.5	5	4.5	4	3.5	3.5	4	4.5	4.5	4
	Std	3.2	3.5	5.1	4.5	4.5	4.2	3.7	3.8	4.8	5.9	5.2	4.8	3.4	3.6	5	5.1	4.7	4.5

**Surgical Admissions**	Mean	0.09	0.14	0.14*	0.14	0.1	0.12	0.09	0.11	0.20*^†^	0.17	0.12	0.14	0.09	0.13	0.17	0.15	0.11	0.13
	Median	0	0	0	0	0	0	0	0	0	0	0	0	0	0	0	0	0	0
	Std	0.27	0.38	0.32	0.37	0.3	0.33	0.31	0.24	0.45	0.42	0.32	0.36	0.28	0.33	0.38	0.39	0.3	0.35

**Hospital Admissions**	Mean	0.05	0.08	0.12^†^	0.17^‡^	0.17^‡^	0.12	0.1	0.08	0.17	0.14	0.22	0.13	0.07	0.08	0.15	0.16	0.19	0.13
	Median	0	0	0	0	0	0	0	0	0	0	0	0	0	0	0	0	0	0
	Std	0.2	0.24	0.3	0.41	0.38	0.32	0.32	0.2	0.34	0.33	0.45	0.32	0.26	0.23	0.32	0.38	0.4	0.32

**Health Conditions**	Mean	6.5	7	8.8^‡^	8.8^‡^	8.8^‡^	7.8*	6.2	6.5	8.0^‡^	8.4^‡^	8.5^‡^	7.4*	6.4	6.8	8	8.6	8.7	7.7
	Median	6	6	8	8	9	7	6	6	7	8	8	7	6	6	8	8	8	7
	Std	3.7	3.7	4.2	4.2	4.8	4.1	3.6	3.5	4.4	4.6	4.1	4.2	3.7	3.6	4	4.3	4.6	4.1

**Medications**	Mean	6.9	7.6*	9.6^‡^	9.6^‡^	9.4^‡^	8.4*	6.2	6.7*	8.8^‡^	9.1^‡^	8.4^†^	7.8*	6.6	7.2	8.7	9.4	9.1	8.2
	Median	6	7	8	8	8.5	8	5.5	6	7	8	7	7	6	7	8	8	8	7
	Std	4.6	4.5	5.6	5.6	5.5	5.1	4.2	4.3	5.6	5.7	5	5.1	4.4	4.5	5.1	5.6	5.3	5.1

*differences between genders at P < 0.05

^†^, ^‡ ^differences between a particular age group and the 65-69 group within a gender group at P < 0.05 and P < 0.001 levels, respectively.

Family practice visits comprised the greatest number of physician services with an average of 4.4 visits per person per year. The oldest group made 46% more visits than the youngest group. Women made 11% more visits than men.

The average rate of specialist visits at 2.4 visits per year was slightly more than half the number of family practice visits. There was an inverted U-shape, with the highest rates of utilization occurring in the 75-84 age groups. Men made 17% more visits than women.

There was an average of 0.22 ER visits per year per patient. The oldest age group visited the ER 131% more often than the youngest age group. There was no statistical difference between men and women.

Overall, there were 0.13 surgical admissions per year per patient. There was an absence of a clear age-related pattern for utilization of this service (only men 75-79 had statistically more visits than the comparison group of men aged 65-69). There was no overall difference between men and women.

There were 0.13 additional annual inpatient hospital admissions per patients not related to surgeries. The oldest age group had 162% more admissions than the youngest group. However, there were no significant gender-related differences in hospitalizations.

There was an average of 7.7 chronic health conditions per patient recorded in the CPP and most recent two years of chart records. The oldest age group had 37% more chronic health conditions than the youngest group. The intra-group variability was generally greater in the older groups. Women had 6% more chronic health conditions than men.

Patients used an average of 8.2 prescription and non-prescription medications over the two year study period. The oldest age group had 37% more different medications recorded in their charts than the youngest group. Again, the intra-group variability was greater in the three older age groups than the younger group. Women used an 8% greater number of medications than men.

## Discussion

Significant differences between the oldest and youngest age groups were observed for FP visits, ER visits, inpatient hospital admissions, test days, and number of chronic health conditions and medications from the patients' CPP. The other indicators, specialist visits and surgeries, did not show age-related differences. Our findings also show that variability within age groups increases with age but decreases in the oldest (85+) group. With regards to gender, women make more FP visits, have more chronic health conditions, and take a greater number of medications, but visit specialists less frequently than men.

### Strengths and Limitations Of This Study

The strengths of this study consist in its detailed analysis of individual data in a family practice setting as well as close examination in age differences in late life course. By analyzing patterns across narrowly-defined age groups, our results have shown that not only does the number of chronic health conditions differ among age groups, but that there is variation *within *age groups as well. A further strength of this study is the lack of potential for sampling bias, as this was a comprehensive chart review of all patients 65 years and older in the clinic.

This study faces the same limitations of any study that relies on chart audit in that the thoroughness of chart documentation may vary considerably among the clinicians, and thus the potential for missing information both in the charting process and the auditing process. In particular, reports for services performed outside of the family practice, or visits to other primary care clinics, may never make their way back to the patient chart. These considerations lead us to believe that our observed measurements are underestimates of the true values.

Another potential limitation to the generalizability of our results relates to the high socioeconomic demographic of the study population in a research based primary care practice due to the location of the clinic in a highly educated and high SES Toronto neighborhood. However, in a province with a highly socialized medical sector, this characteristic may play less of a role. The maximum difference in visit rates between seniors of different income quintiles is 12% for Ontario seniors [14]. Moreover, it has been shown that research in research active primary care practices are generalizable to those that do not participate in research [15]. Our study population also did not include elderly moving on to extended care facilities (such as nursing homes) and may have severe functional limitations. While this is an important outcome to consider, this data is not reliably available from the chart. This further limits the generalizability of our study as our study only includes ambulatory patients living at home and visiting the HSO.

### Interpretation

Our results generally support a theme of increasing utilization among a proportion of patients with advancing age, reflecting more diverse health care needs with age. This is consistent with population based data indicating similar patterns in the province of Ontario [14,16,17]. In previous studies, age has been associated with higher levels of health service utilization [12]. However, in a study specific to individuals over 75 years old, age was not significantly associated with healthcare utilization [13]. Our result is consistent with both pictures; we used a younger age group (65-69) as our baseline and we too observed leveling or declining use with our oldest group. Potential explanations for the declining use of physician services among the oldest adults include institutional substitution, informal substitution, losing a longstanding physician, developing stabilized regimens, the patient giving up, or the doctor giving up [18]. A more likely explanation could be that those people surviving into the oldest age group were healthier and thus more likely to have been low users of health care services to begin with. This is further supported by our results pertaining to variability within age groups.

Heterogeneity or variability within an age group has been shown to increase with age in a variety of fields [19]. This study adds further evidence from a primary care practice that health service utilization, medication use, and chronic health conditions follow this trend. Previous studies have found that most contacts of high users of health care are for treatment and follow-up of chronic diseases. High users have also reported significantly more medical conditions of all types and perceived themselves to have poorer health status [20,21]. Our results showed that there was generally either a leveling off or a decrease in both mean utilization rates and standard deviation of the 85+ age group from the 80-84 age group.

The average life expectancy in Canada is 84 for women and 82 for men,[1] and as health care utilization often peaks near the end of life [10], it could be expected that the 80-84 group would have higher mean utilization and greater variability than the oldest group. The relative health of the oldest group has been explained by aging theories pertaining to resilience [22]. aging trajectories [23,24], and profiles of 'survivors, escapers, and delayers'[25], which all describe how morbidity in the late life course can follow different patterns.

Our gender-related results showing that women make more FP visits, have more chronic health conditions, take more medications, but make fewer visits to specialists are broadly consistent with previous studies. Women have been previously shown to use more primary care and diagnostic services than men [6,7,11-13], and those after age 75 have a greater number of chronic conditions than men [7,22,25]. The trends for other medical services, such as specialist usage, emergency room visits, hospitalizations, and surgeries have been mixed, with some results showing that men had greater utilization [7,8], and some showing no differences [6,11,12].

Our findings could be interpreted to support a hypothesis about gender and healthcare that on average, men delay prevention and maintenance services which could result in greater need for downstream treatment [26] but that the trends and underlying explanations are not entirely clear and may need periodic revisiting [7,9]. It also could represent a gender bias in access to specialized health services, and could be part of a troubling gender equity bias in Ontario [27-29].

### Implications

The major policy implication arising from our observations is a forthcoming increase in the demand for primary care and ER services relative to specialist consultants and surgeons. While a shortage of physicians is anticipated in all fields, primary care should be considered a priority for expansion of post-graduate training programs.

Secondly, clinical strategies for seniors must be re-evaluated. While studies including ours have found that the number of chronic health conditions is higher among older adults, the length of time of physician visits has not been found to be significantly different from middle-aged adults [30]. The higher numbers of chronic health conditions also mean that following disease-specific guidelines might not only be impractically time consuming [3], but also lead to unwanted side-effects [4]. While targeted disease-management programs for conditions such as diabetes and heart disease are still required, future improvements to primary care practice should take the patient-centered approach, which focus on prevention, health promotion, and individual needs of older patients who vary on the wellness-illness continuum. An interprofessional team-based method of multiple chronic disease management in this patient population may be a more optimal approach, as individuals in the highest quartile of events are poorly served by episodic care, short visit times, and strategies of managing complexity that yield more utilization. Also, multiple chronic illnesses requires patients and care-givers to navigate among many different health care professionals, where barriers to the transmission of health information and differences of focus in the plan of care among health care professionals may occur. To that end, the IMPACT clinic (Interprofessional Model of Practice for Aging & Complex Treatments) was started in the Family Practice Unit at Sunnybrook Health Sciences Centre to evaluate effectiveness and feasibility of a team based approach to chronic disease management [5]. Further, this points to a stronger role for medical schools and other health professional training programs in developing a diverse skill set in prevention and treatment of complex health profiles and dispelling prejudices about the latter part of the life course. These efforts could have downstream benefits in reducing the need for scarce and costly treatments and increase the prevalence of 'delayers' and 'escapers', or in other terms, rectangularize the morbidity curve.

## Conclusion

In conclusion, with the exception of the oldest age group, we found higher utilization and greater intra-group variability with advancing age. This study should inform the development of benchmark indicators, but further work is needed to build on the data we present. It remains a priority for further investigation whether greater utilization of services and medications is linked to better outcomes in morbidity, mortality, and quality of life for patients. Finally, there is the need to create and evaluate innovative models of care of multiple chronic conditions in the late life course.

## Competing interests

The authors declare that they have no competing interests.

## Authors' contributions

KV led the statistical analysis the data, participated in the literature review, drafted the first version of the manuscript, and contributed to subsequent revisions. JXN participated in the design of the study, coordinated the data collection process, assisted with the literature review, participated in the data analysis, and contributed to the drafting and revising of the manuscript. LW participated in the design of the study, assisted with the literature review, participated in the data analysis, and contributed to the drafting and revising of the manuscript. CST participated in the design of the study, participated in the data analysis, and contributed to the drafting and revising of the manuscript. RM participated in the design of the study, participated in the data analysis, and contributed to the drafting and revising of the manuscript. REGU conceived the idea for the study, participated in study design, contributed to the data analysis and to the drafting and revising of the manuscript, and will act as study guarantor. All authors have read and approved the final manuscript.

## Pre-publication history

The pre-publication history for this paper can be accessed here:

http://www.biomedcentral.com/1472-6963/9/217/prepub
